# Supplementary material for: Understanding clinician connections to inform efforts to promote high-quality inflammatory bowel disease care
Source: PLoS One. 2022 Dec 27;17(12):e0279441. doi: 10.1371/journal.pone.0279441 (PMC9794045; doi:10.1371/journal.pone.0279441)
Supplement: S1 Table — (DOCX) [file pone.0279441.s001.docx]

| **Supplemental Table 1. Measures of centrality and associated provider characteristics for GI providers only** | | | | | | |  |
| --- | --- | --- | --- | --- | --- | --- | --- |
| **Provider characteristic** | **Total** | **Degree (mean, sd)** | **P-value** | **Closeness (mean, sd)** | **P-value** | **Betweenness (mean, sd)** | **P-value** |
| Type of provider (MD, NP, PA) |  |  | 0.5259 |  | 0.0895 |  | 0.5686 |
| NP | 284 (17.16%) | 23.63 (25.34) |  | 51.11 (24.41) |  | 217.59 (467.15) |  |
| Physician | 1231 (74.38%) | 22.85 (25.31) |  | 51.01 (23.63) |  | 227.99 (632.84) |  |
| PA | 140 (8.46%) | 25.32 (27.3) |  | 55.66 (24.62) |  | 282.23 (729.35) |  |
| Number of IBD patients (quartile) |  |  | 0.0000 |  | 0.0000 |  | 0.0000 |
| < 4 | 414 (25.02%) | 3.59 (2.78) |  | 36.96 (19.63) |  | 2.74 (9.87) |  |
| < 13 | 414 (25.02%) | 10.38 (5.1) |  | 46.34 (18.81) |  | 40.46 (98.29) |  |
| < 34 | 414 (25.02%) | 21.37 (9.47) |  | 53.17 (20.59) |  | 167.3 (536.28) |  |
| >= 34 | 413 (24.95%) | 57.52 (27.34) |  | 69.27 (23.73) |  | 713.85 (949.4) |  |
| Number of IBD visits (quartile) |  |  | 0.0000 |  | 0.0000 |  | 0.0000 |
| < 5 | 414 (25.02%) | 3.62 (2.81) |  | 36.91 (19.29) |  | 3.97 (15.85) |  |
| < 21 | 414 (25.02%) | 11.8 (5.95) |  | 46.52 (19.54) |  | 57.38 (196.29) |  |
| < 59 | 414 (25.02%) | 21.87 (11.47) |  | 53.59 (19.71) |  | 176.81 (530.31) |  |
| >= 59 | 413 (24.95%) | 55.55 (29.41) |  | 68.72 (24.54) |  | 686.1 (955.31) |  |
| Proportion of Crohn's |  |  | 0.0000 |  | 0.0000 |  | 0.0000 |
| < 25% | 414 (25.02%) | 9.63 (11.12) |  | 41.31 (21.58) |  | 53.9 (138.21) |  |
| < 35.4% | 414 (25.02%) | 28.26 (23.53) |  | 55.44 (23.49) |  | 343.79 (836.42) |  |
| < 46.1% | 414 (25.02%) | 38.79 (31.41) |  | 61.51 (24.27) |  | 375.85 (613) |  |
| >= 46.1% | 413 (24.95%) | 16.07 (20.8) |  | 47.43 (20.98) |  | 149.43 (597.38) |  |
| Proportion of UC |  |  | 0.0000 |  | 0.0000 |  | 0.0000 |
| < 25% | 414 (25.02%) | 16.96 (21.91) |  | 48.69 (22.83) |  | 161.19 (592.58) |  |
| < 50% | 414 (25.02%) | 35.08 (31.86) |  | 58.5 (25) |  | 332.1 (598.73) |  |
| < 75% | 414 (25.02%) | 30.56 (24.19) |  | 56.25 (23.55) |  | 379.32 (853.44) |  |
| >= 75% | 413 (24.95%) | 10.13 (10.71) |  | 42.24 (20.42) |  | 50.14 (120.46) |  |
| Proportion of indeterminate |  |  | 0.0000 |  | 0.0000 |  | 0.0000 |
| <= 0% | 414 (25.02%) | 7.09 (6.48) |  | 40.53 (18.32) |  | 19.83 (70.03) |  |
| < 7.7% | 414 (25.02%) | 22.4 (23.13) |  | 50.27 (23.15) |  | 201.75 (439.89) |  |
| < 13.7% | 414 (25.02%) | 40.02 (28.8) |  | 61.35 (24.65) |  | 491.15 (947.35) |  |
| >= 13.7% | 413 (24.95%) | 23.26 (25.52) |  | 53.55 (24.11) |  | 210.39 (559.83) |  |
| Location of practice |  |  | 0.0066 |  | 0.0000 |  | 0.2853 |
| Rural | 61 (3.72%) | 14.59 (14.82) |  | 31.47 (12.65) |  | 147.06 (259.72) |  |
| Urban | 1577 (96.28%) | 23.65 (25.84) |  | 52.33 (23.92) |  | 233.35 (628.31) |  |
| Facility complexity |  |  | 0.0000 |  | 0.0000 |  | 0.0009 |
| 1a | 835 (50.45%) | 26.16 (29.54) |  | 57.17 (22.32) |  | 226.34 (523.56) |  |
| 1b | 425 (25.68%) | 21.7 (22.75) |  | 55.83 (26.35) |  | 324.26 (936.91) |  |
| 1c | 266 (16.07%) | 20.36 (17.71) |  | 37.25 (15.32) |  | 140.68 (226.19) |  |
| 2 | 101 (6.1%) | 13.85 (13.34) |  | 30.45 (10.94) |  | 122.34 (236.71) |  |
| 3 | 28 (1.69%) | 17.93 (14.47) |  | 23.47 (10.32) |  | 192.1 (247.57) |  |
| Region |  |  | 0.0015 |  | 0.0000 |  | 0.031 |
| Continental | 235 (14.35%) | 22.16 (24.98) |  | 50.88 (21.21) |  | 205.99 (408.08) |  |
| Midwest | 405 (24.73%) | 22.85 (25.3) |  | 59.88 (28.29) |  | 307.29 (907.42) |  |
| North Atlantic | 405 (24.73%) | 23.38 (26.02) |  | 44 (21.14) |  | 182.08 (535.11) |  |
| Pacific | 267 (16.3%) | 19.34 (21.85) |  | 44.41 (18.12) |  | 187.12 (404.18) |  |
| Southeast | 326 (19.9%) | 27.9 (27.94) |  | 56.93 (22.62) |  | 246.62 (531.51) |  |
| *univariable (reported as centrality measures and ANOVA tests) | | | | | | | |
